# Supplementary material for: Herbal Compounds Dauricine and Isoliensinine Impede SARS-CoV-2 Viral Entry
Source: Biomedicines. 2023 Oct 27;11(11):2914. doi: 10.3390/biomedicines11112914 (PMC10669532; doi:10.3390/biomedicines11112914)
Supplement: Supplementary file 1 [file biomedicines-11-02914-s001.zip › biomedicines-2654942-SI.pdf]

Supplementary Figures and Tables

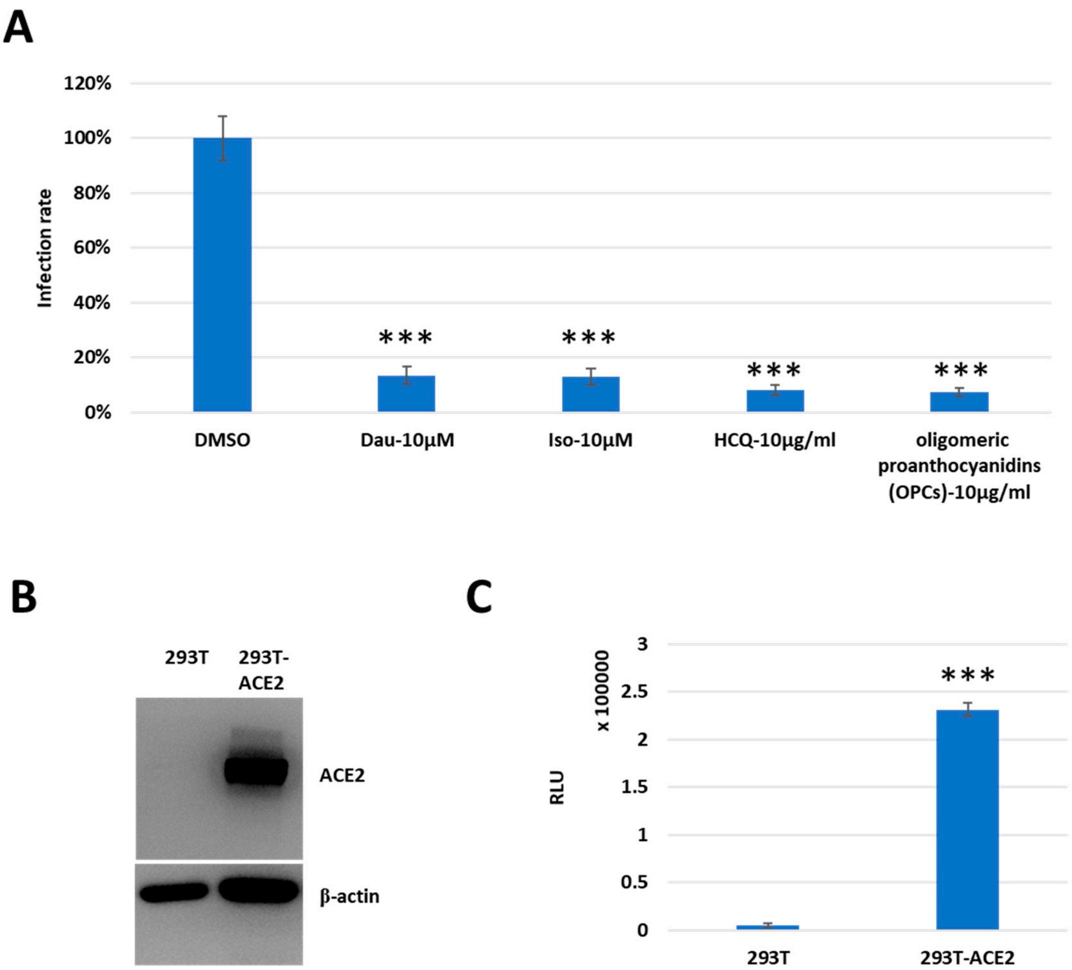

Supplemental Figure S1. The effect of ACE2 overexpression on infection rates. (A) 293T-ACE2 cells were pretreated with the indicated concentration of Dauricine, Isoliensinine, Hydroxychloroquine (HCQ), or oligomeric proanthocyanidins (OPCs) for one hour and infected with SARS-CoV-2 WT-VPP. After 24 hours of infection, the infection efficiency rate was measured according to luciferase activities. Values are normalized to vehicle control (100%) and shown as mean ± SD (n=3). ***, P<=0.001 compared to vehicle control. (B) Western blot of ACE2 in 293T expressing ACE2 or parental cells. (C) 293T cells with and without ACE2 expression were infected with SARS-CoV-2 WT-VPP. After 24 hours of infection, the infection efficiency was measured according to luciferase activity (relative light units (RLU)). ***, P<=0.001 compared to the 293T group.

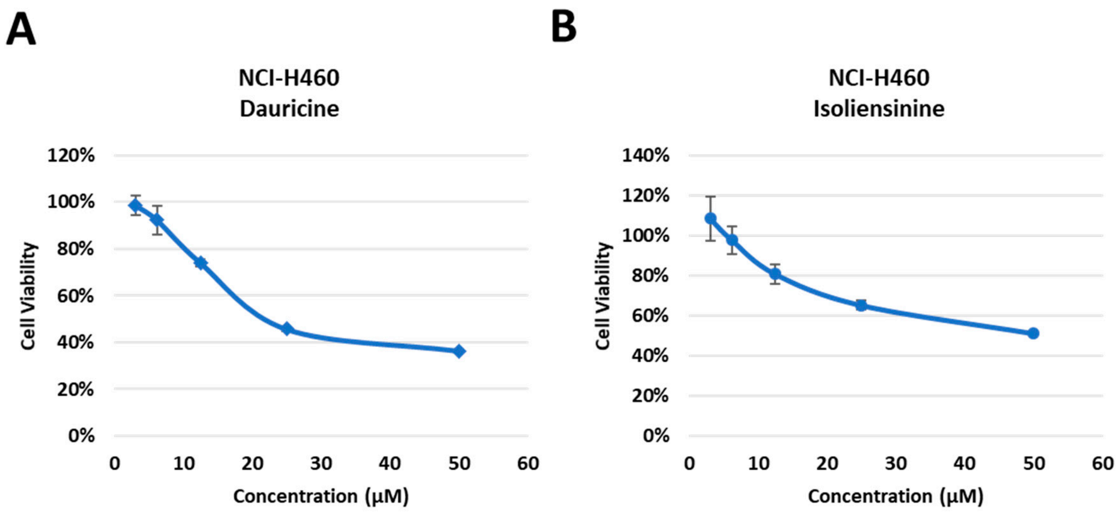

Supplemental Figure S2. Cytotoxic activity of Dauricine and Isoliensinine in NCI-H460 cells. (A) and (B) NCI-H460 cells were treated with different concentrations (3.125, 6.25 12.5, 25 and 50 μM) of Dauricine (A) or Isoliensinine (B), and cell viability was detected by MTT assay. Values are normalized to vehicle control (100%) and shown as mean ± SD (n=3).

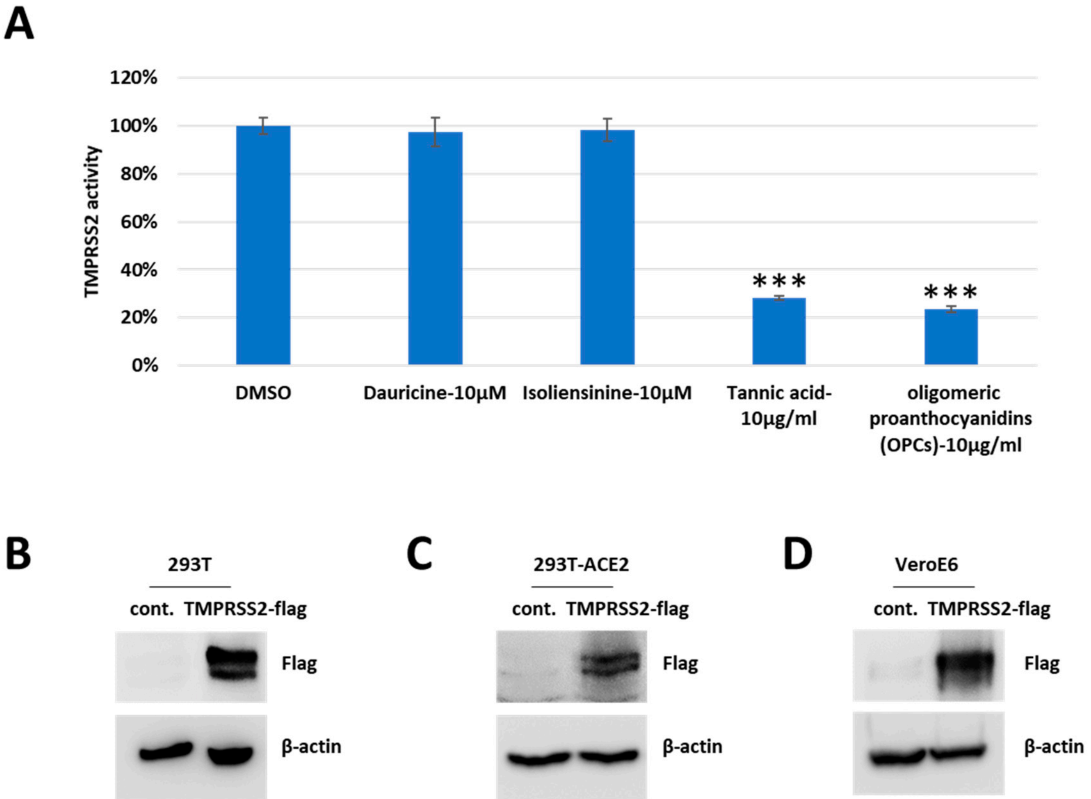

Supplemental Figure S3. The effect of Dauricine and Isoliensinine on TMPRSS2 activity. (A) The TMPRSS2 enzymatic activity in vivo was measured by using a FRET-base assay with indicated concentration of Dauricine, Isoliensinine, tannic acid or oligomeric proanthocyanidins (OPCs). Values are normalized to vehicle control (100%) and shown as mean ± SD (n=3). ***, P<=0.001 compared to vehicle control.(B) Western blot of TMPRSS2-Flag in 293T expressing TMPRSS2 or vector control cells. (C) Western blot of TMPRSS2-flag in 293T-ACE2 expressing TMPRSS2 or vector control cells. (D) Western blot of TMPRSS2-Flag in VeroE6 expressing TMPRSS2 or vector control cells.

Supplemental Table S1. The distance and main interactive mode of each amino acid residue with Dauricine. Protein residue interactions corroborated using Pymol.

Variant	Amino acid	Distance (Å)	Interactive mode
B.1.1.7	ARG-408	3.4	Hydrogen bond
	ARG-403	3.5	Hydrogen bond
	ALA-387	3.1	Hydrogen bond
B.1.351	ASN-33	3.1	Hydrogen bond
	ALA-387	3.2	Hydrogen bond
	ARG-393	3.2	Hydrogen bond
P.1	ASN-33	2.9	Hydrogen bond
	ALA-387	3.3	Hydrogen bond
B.1.617	PHE-390	3.0	Hydrogen bond
	ASN-33	3.5	Hydrogen bond
B.1.529	HIS-505	2.7	Hydrogen bond
	ARG-403	3.1	Hydrogen bond
	ASN-417	3.0	Hydrogen bond
	ARG-408	2.8	Hydrogen bond
Wild Type (WT)	ARG-457	3.1	Hydrogen bond

Supplemental Table S2. The distance and main interactive mode of each amino acid residue with Isoliensinine. Protein residue interactions corroborated using Pymol.

Variant	Amino acid	Distance (Å)	Interactive mode
B.1.1.7	Arg403	2.7	Hydrogen bond
	Arg403	2.4	Salt bridge
	Asp30	3.5	Hydrogen bond
B.1.351	Arg393	2.9	Hydrogen bond
	Arg393	3.1	Salt bridge
P.1	His34	3.4	Hydrogen bond
	Arg393	3.1	Hydrogen bond
	Ala386	3.4	Hydrogen bond
B.1.617	Glu37	3.3	Hydrogen bond
	Arg403	3.3	Hydrogen bond
	Arg403	2.7	Hydrogen bond
	His34	3.1	Hydrogen bond
	His34	3.1	Salt bridge
B.1.529	Asn417	2.6	Hydrogen bond
	Arg403	3.4	Hydrogen bond
	Arg403	3.4	Hydrogen bond
	Pro389	3.5	Hydrogen bond
Wild Type (WT)	Ser514	2.7	Hydrogen bond
	Arg355	2.4	Salt bridge
	Arg355	3.3	Salt bridge
	Arg355	3.5	Hydrogen bond
